# Effect of hookworm infection and anthelmintic treatment on naturally acquired antibody responses against the GMZ2 malaria vaccine candidate and constituent antigens

**DOI:** 10.1186/s12879-021-06027-5

**Published:** 2021-04-08

**Authors:** Benjamin Amoani, Ben Gyan, Samuel Asamoah Sakyi, Emmanuel Kwasi Abu, Samuel Victor Nuvor, Precious Barnes, Tracy Sarkodie-Addo, Benjamin Ahenkorah, Christian Sewor, Duah Dwomoh, Michael Theisen, Michael Cappello, Michael D. Wilson, Bright Adu

**Affiliations:** 1 Department of Biomedical Science, School of Allied Health Sciences, University of Cape Coast, Cape Coast, Ghana; 2 Department of Molecular Medicine, School of Medical Sciences, Kwame Nkrumah University of Science and Technology, Kumasi, Ghana; 3 Department of Immunology, Noguchi Memorial Institute for Medical Research, College of Health Sciences, University of Ghana, Legon, Ghana; 4 Department of Optometry, School of Allied Health Sciences, University of Cape Coast, Cape Coast, Ghana; 5 Department of Microbiology and Immunology, School of Medical Sciences, University of Cape Coast, Cape Coast, Ghana; 6 Department of Physician Assistant, School of Allied Health Sciences, University of Cape Coast, Cape Coast, Ghana; 7 Department of Medical Laboratory Science, Bolgatanga Technical University, Bolgatanga, Upper East Region Ghana; 8 Department of Biostatistics, School of Public Health, University of Ghana, Accra, Ghana; 9 Department for Congenital Disorders, Statens Serum Institut, Copenhagen, Denmark; 10 Centre for Medical Parasitology at Department of International Health, Immunology, and Microbiology, University of Copenhagen, and Department of Infectious Diseases, Copenhagen University Hospital, Rigshospitalet, Copenhagen, Denmark; 11 Partnerships for Global Health, Department of Pediatrics, Yale School of Medicine, Yale University, New Haven, CT USA; 12 Parasitology Department, Noguchi Memorial Institute for Medical Research, College of Health Sciences, University of Ghana, Legon, Ghana

## Abstract

**Background:**

Malaria and helminths diseases are co-endemic in most parts of sub-Saharan Africa. Immune responses from each of these pathogens interact, and these interactions may have implications on vaccines. The GMZ2 malaria vaccine candidate is a fusion protein of *Plasmodium falciparum* merozoite surface protein 3 (MSP3) and glutamate rich protein (GLURP R0). GMZ2 has recently showed modest efficacy in a phase IIb multicenter trial. Here, we assessed the effect of hookworm (*Necator americanus*) infection and anthelmintic treatment on naturally acquired antibody responses against GMZ2 and constituent antigens.

**Methods:**

This longitudinal cross-sectional study was conducted in the Kintampo North Municipality of Ghana. Blood and stool samples were taken from 158 individuals (4–88 years old) infected with either *P. falciparum* alone (*n* = 59) or both hookworm and *P. falciparum* (*n* = 63) and uninfected endemic controls (*n* = 36). Stool hookworm infection was detected by the Kato-Katz method and PCR. Malaria parasitaemia was detected by RDT, light microscopy and *P. falciparum*-specific 18S rRNA gene PCR**.** Serum samples were obtained prior to hookworm treatment with a single dose of albendazole (400 mg) and 3 weeks (21 days) after treatment. Levels of IgG1, IgG3 and IgM against GMZ2, MSP3 and GLURP R0 were measured by ELISA and compared among the groups, before and after treatment.

**Results:**

Participants with *P. falciparum* and hookworm co-infection had significantly higher IgG3 levels to GMZ2 than those with only *P. falciparum* infection and negative control (*p* < 0.05) at baseline. Treatment with albendazole led to a significant reduction in IgG3 levels against both GMZ2 and GLURP R0. Similarly, IgM and IgG1 levels against MSP3 also decreased following deworming treatment.

**Conclusion:**

Individuals with co-infection had higher antibody responses to GMZ2 antigen. Treatment of hookworm/malaria co-infection resulted in a reduction in antibody responses against GMZ2 and constituent antigens after albendazole treatment. Thus, hookworm infection and treatment could have a potential implication on malaria vaccine efficacy.

## Introduction

Malaria and hookworm are important parasitic diseases in humans, in terms of socio-economic impact and public health importance. Malaria is typically an acute disease; however, chronic asymptomatic infections may persist, particularly in adults living in endemic areas. Hookworm infection on the other hand is mainly chronic. In areas where these parasites are co-endemic and the transmission rates are high, co-infection is very common and this may influence morbidity and immune response against these infections [1–4], with possible implications on vaccine efficacy. The GMZ2 malaria vaccine candidate is a recombinant fusion protein, containing two blood-stage antigens of *P. falciparum*, GLURP R0 and MSP3 [5]. Previous phase 2 clinical trial of GMZ2 showed it well tolerated and immunogenic albeit with modest efficacy [5]. This is not uncommon since most malaria vaccine candidate efficacy trials in Africa has so far not been remarkably efficacious against malaria [6–11]. However, factors modulating immune responses against malaria vaccine candidates that may help provide possible explanations to the sub-optimal efficacies observed are not well understood. Helminths secrete immunomodulatory molecules to selectively skew or dampen immune responses to promote their long-term survival [12]. Hookworm infection is associated with higher levels of IL-10 and lower levels of both Th1 and Th2 cytokines [13]. The enhanced IL-10 production may be a mechanism to regulate pathology due to inflammatory responses elicited by the infection Hookworm infection is very common in Africa, however its effect on malaria vaccine candidate antigens such as GMZ2 have not been extensively investigated.

Treatment of infected people with anthelmintic drug may alter antimalarial specific immune responses [1, 2, 14, 15]. Since albendazole treatment is administered to hookworm infected people who may be living in malaria endemic communities (exposed to both malaria and hookworm), it is important to determine if this chemotherapy alters any of the responses to malaria vaccine candidate antigens and inadvertently affect vaccine efficacy. We have previously shown that in the absence of other helminths, co-infection of hookworm with *P. falciparum* may modulate blood parasitemia levels and cytokine responses [16]. Different helminths modulate the immune response to malaria differently. *Trichuris trichiura* infections in malaria patients was associated with reduced antibody levels against *P. falciparum* antigens [3]. Similarly, Courtin et al., [17] found *Schistosoma haematobium* infection to be associated with reduction in IgG levels against *P. falciparum* antigens MSP-1 and GLURP. Gabonese children infected with *Ascaris lumbricoides* had a significantly higher *P. falciparum* anti-gametocyte antibody levels compared to non-infected children [4]. Interestingly, *T. trichiura* infection was associated with lower *P. falciparum* anti-gametocyte antibody levels in the same study [4]. To date, hookworm modulation of the antibody responses against malaria vaccine antigens has not been explored. Thus, the current study aimed to investigate the effect of hookworm infection and anthelmintic treatment on antibody responses against the GMZ2 vaccine and its constituent antigens in a population co-endemic with both parasites*.*

## Materials and methods

### Ethics approval and consent to participate

The study was approved by the Noguchi Memorial Institute for Medical Research Ethical Review Committee (FWA#: 00001824). All methods were carried out in accordance with relevant guidelines and regulations.

### Study site, design, and sample processing

The study was conducted from February to March in 2016, in communities within the Kintampo North Municipality (KNM) in the forest-savannah transitional ecological zone of middle Ghana. The KNM covers a total area of 7162 km2 with a population of approximately 140,000 in 32,329 households. The inhabitants are predominantly subsistent farmers of both crop and livestock. The study involved baseline sampling and a follow-up at 3 weeks post-anthelmintic treatment, a durbar was first held in each study village during which the purpose and the nature of the study were explained. A total of 1068 potential study participants aged 4–88 years were randomly identified from a population census data base and recruited into the study. Study subjects (*n* = 984) who appeared healthy and were without fever were consented individually prior to providing stool and blood samples [16, 17]. Briefly, trained field workers administered a demographic and health questionnaire, and distributed labelled stool-collection containers to the participants. Stool samples were collected the following day, and finger pricks were made to test for malaria using Rapid Diagnostic Test (RDT) kits and to prepare thin and thick blood film slides. Malaria parasitaemia was also detected by light microscopy and *P. falciparum*-specific 18S rRNA gene PCR [16]**.** Stool hookworm infection was detected by the Kato-Katz method and PCR for speciation [16]. About 5 ml of venous blood was collected by venipuncture using Becton Dickinson Vacutainer Hemogard SST® tubes (Becton Dickson and Company, Temse, Belgium) for serum separation from study participants). Blood samples were obtained prior to hookworm treatment with a single dose of albendazole (400 mg) and 3 weeks (21 days) after treatment. The blood sample were separated by centrifugation, and the serum were stored at − 80 °C until ready to be used for the immunological assays.

### Malaria antibody measurement in sera

Serum antibodies to the malaria antigens (MSP3, GLURP and GMZ2) were measured using a modified version of a quantitative ELISA [18]. GLURP and MSP3 are components in the developmental stages of *P. falciparum* [19]. GLURP is an *Escherichia coli* recombinant protein containing the conserved non-repeat N-terminal region (amino acids 25–514) [20]. The MSP3 antigen is a long synthetic peptide called LR55 (amino acids 181–276) of the merozoite surface protein 3 [21]. GMZ2, contains conserved fragments of two *P. falciparum* asexual blood-stage antigens, Glutamate-Rich Protein (GLURP) and MSP3 [5]. Briefly, ELISA plates (Nunc, Maxisorp, Fisher Scientific, USA) were coated with 0.5 μg/ml of the recombinant antigens. Individual and positive control serum samples were diluted at 1:200 for IgM and 1:100 for IgG1and IgG3. Bound antibodies were detected by horse-radish conjugated sheep anti-human IgG1, and IgG3 (1:1000) (The Binding Site, Birmingham, UK) or goat anti-human IgM (1: 5000) (Invitrogen, USA). Color development was by TMB (3,3′,5,5′-tetramethylbenzidine) substrate (Kem-En-Tec Diagnosis A/S, Taastrup, Denmark) and the reaction stopped with 0.2 M H_2_SO_4_. Optical density (OD) values were read at 450 nm with a reference wavelength of 570 nm with an automated ELISA reader (BioTek 405, USA). The OD values obtained were converted into arbitrary units (AU) using the four-parameter curve fitting software (ADAMSEL, version 1.1 build 40© 2009 EJ Remarque). The two time point samples for each individual were tested on the same ELISA plate to avoid differences that may have been due to inter-plate variations.

### Statistical analyses

Data analysis was performed using R version 3.6. 0 (https://www.Rproject.org/). Arbitrary antibody units (AU) were transformed into Log_10_ units. Linear regression was used to determine the association between antibody level and age. Association between antibody levels and infection status was assessed by multivariate regression analysis adjusting for age and sex with the endemic negative control group as the reference. Wilcoxon signed ranks test was used to assess if there was any significant difference in antibody levels before and after albendazole treatment. *P* values < 0.05 were considered statistically significant.

## Results

### Demographic and clinical characteristics of study participants

A prevalence of 10.5% (103/984) for hookworm infection and 12.4% (122/984) for *P. falciparum* infection were observed [16]. Of these subjects who were infected with malaria; 59 were only *P. falciparum* (*Pf*), and 63 were co-infected with both hookworm and *P. falciparum* (*Na/Pf*). Thirty-six uninfected subjects were randomly selected to serve as uninfected (EC) assay controls (*n* = 36). The mean age of 16.2 years (yrs.), 25.5 yrs., 36.3 yrs. were recorded for *Pf, Na/Pf* and EC, respectively for the participants [22]. *P. falciparum* parasite density was significantly lower in the co-infected group than in those singly infected with only *P. falciparum*, as indicated in Table 1. Other soil transmitted helminths observed by microscopy were either present as mono-infections: *Hymenolepis nana* (Hn) (3.9%), *Taenia solium* (Ts) (0.8%), *Trichuris trichiura* (Tt) (1.8%) and *Ascaris lumbricoides* (Al) (0.5%) or coinfections with hookworm (Na/Hn = 1.1%; Na/Ts = 0.3%; Na/ Tt = 0.6%; Na/Al = 0.3%) [16]. Subjects with these helminths, either as mono-infections or co-infections with Na were excluded from further analysis. PCR analysis confirmed all hookworm infections were due to only *Necator americanus* (Na) and none to be *A. duodenale* [16]*.*

**Table 1:** Socio-demographic characteristics of the study population

	***P. falciparum*** only (59)	***Na*** - ***Pf*** (co-infected)(63)	***EC group*** (36)	***Na only***	***P-value***
Male n (%)	20 (33.9)	43 (68.3)	16 (44.4)	17 (42.5)	–
Female n (%)	39 (66.1)	20 (31.7)	20 (55.6)	23 (57.5)	
Age (Mean ± S.D)	16.2 ± 14.26	16.19 ± 10.55	36.32 ± 19.27	31.88 ± 18.71	< 0.0001^a^
Pf density (Mean ± S.D)	1481 ± 3263	794.4 ± 2021	–	–	0.1621^b^

^a^ Analysis was done using ANOVA; ^b^ Analysis was done using Unpaired T-test. Analysis was done only on Pre-treatment data

### Association between anti-GMZ2, MSP3 and GLURP R0 antibody levels and age

**Fig. 1:**
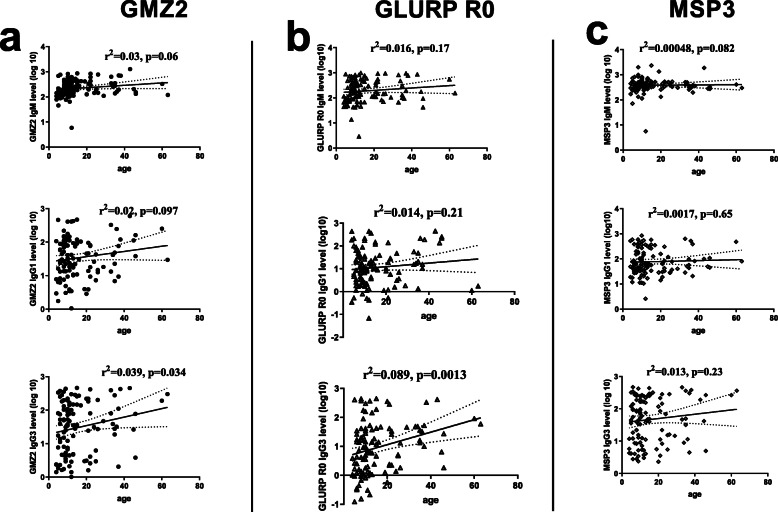
Isotype IgM and IgG sub-class levels in relation to age. Plots are shown (from top to bottom) for IgM, IgG1 and IgG3, levels against age (years), respectively for: **a** GMZ2, **b** GLURP R0 and **c** MSP3. *r*^2^ and *p*-values obtained from linear regressions are shown for each plot. Analysis was done for individuals who were single infected with *P, falciparum* or coinfected with hookworm. Figure 1 analysis was done for the pre-treatment data

The relationship between anti-GMZ2, MSP3 and GLURP R0 antibody levels and age was assessed by linear regression analysis. Increasing age was associated with a significant increase in IgG3 (*r*^2^ = 0.039, *p* = 0.034) against GMZ2 (Fig. 1). There was also a significant increase in IgG3 (*r*^2^ = 0.089, *p* = 0.013) levels against GLURP R0 with age (Fig. 1b). No significant association was observed between MSP3 antibody level and age in this cohort (Fig. 1c). This analysis was done from the pre-treatment data.

### Antibody responses against GMZ2 and constituent antigens among infection groups

Antibody levels against GMZ2, MSP3 and GLURP R0 were compared among individuals with different infection statuses in the study population by Multivariate Multiple Linear Regression MMLR (Table 2). Malaria parasite density had no significant effect in the preliminarily analysis and was therefore excluded from the MMLR analysis. In general, the *N. americanus- P. falciparum* (*Na-Pf*) co-infected individuals had higher antibody levels to all the three antigens compared to the EC individuals. Individuals with *Na-Pf* co-infection had significantly higher IgG3 levels against GMZ2 than the negative endemic control group (*p* = 0.046). Also, IgG1 levels were significantly higher against GLURP-R0 antigens (*p* = 0.019). There was no significant difference in the IgM and IgG1 levels against GMZ2 between the Pf and EC group (*p* > 0.05) or the Na/Pf and EC group (*p* > 0.05). Also, there was no significant difference in the IgM and IgG3 levels against GLURP R0 between the Pf and EC group (*p* > 0.05) or the Na/Pf and EC group (*p* > 0.05). Furthermore, no significant difference was observed for any of the MSP3 antibodies among Pf or Na/Pf groups and the endemic negative control group (Table 2). This analysis was done from the pre-treatment data.

**Table 2:** Antibody responses against GMZ2 and constituent antigens in relation to malaria and hookworm infections

		***P. falciparum*** only		***Na*** + ***Pf*** (co-infected)	
Antibody	Antigen	β (95%CI)	***p***-Value	β (95%CI)	***p***-Value
IgG1	GMZ2	0.01 (−0.66, 0.69)	0.967	−0.41 (−1.08. 0.26)	0.233
	GLURP-R0	0.39 (−0.33, 1.53)	0.184	0.82 (0.31, 1.51)	**0.019**
	MSP3	−0.26 (− 0.80, 0.28)	0.346	0.23 (− 0.32, 0.78)	0.402
IgG3	GMZ2	0.30 (−0.57, 1.18)	0.495	0.3 (1.04, 1.10)	**0.046**
	GLURP-R0	0.26 (−0.61, 1.14)	0.554	0.61 (−0.38, 1.60)	0.226
	MSP3	0.37 (−0.17, 1.72)	0.358	0.24 (−0.53, 1.01)	0.537
IgM	GMZ2	0.13 (−0.26, 0.53)	0.503	0.05 (−.030, 0.40)	0.774
	GLURP-R0	0.08 (−0.66, 0.49)	0.771	0.26 (−0.24, 0.78)	0.300
	MSP3	−0.01 (− 0.32, 0.29)	0.924	0.13 (− 0.22, 0.49)	0.458

Multivariate multiple linear regression analysis adjusting for age and sex. *β* Estimated effect of covariate on antibody level, *CI* Confidence interval, *Na* Na*, Necator americanus, Pf Plasmodium falciparum.* Arbitrary antibody unit were log10 transformed. EC group was set as reference for the model. Analysis was done only on pre-treatment data

### Effect of Albendazole treatment on antibody levels against GMZ2 and constituent antigens in Na/Pf co-infected group

Treating Na/Pf co-infected individuals with albendazole led to a significant reduction of IgG1 (*p* = 0.002) and IgG3 (*p* = 0.041) levels against GMZ2, and IgG3 (*p* = 0.024) levels against GLURP R0. There were also significant reduction in IgM and IgG1 (*p* < 0.001) levels against MSP3 following albendazole treatment in the same individuals (Table 3).

**Table 3:** Effect of albendazole treatment on antibody levels against GMZ2 and constituent in Na/Pf co-infected individuals

Vaccine antigens	Pretreatment (***n*** = 57) (Median [IQR])	Post treatment (***n*** = 57) (Median [IQR])	***P***-value
**GMZ2**			
IgG1	38.2(10.2,90.6)	30.0(11.2,66.8)	**0.002**
IgG3	34.6(4.5, 142.0)	28.3(2.7121.3)	**0.041**
IgM	192.5(137.3326.2)	212.6(106.9400.0)	0.403
**GLURP R0**			
IgG1	8.5(2.6,29.0)	8.2(0.9,24.3)	0.647
IgG3	14.3(2.0,45.4)	13.6(3.00,30.6)	**0.024**
IgM	155.8(88.8375.1)	139.5(81.2, 385.8)	0.184
**MSP3**			
IgG1	56.2(28.5143.8)	41.7(28.0,52.7)	**< 0.001**
IgG3	44.9(8.3167.2)	50.1(23.9, 128.2)	0.130
IgM	332.8(269.2452.8)	36.3(10.6149.2)	**< 0.001**

Values are median (quartile). *P*-values were calculated using the Wilcoxon Signed Ranks Test

This analysis was done for those with hookworm and malaria parasites co-infection; pre-treatment (*n* = 57) and post-treatment (*n* = 57). Six individuals were lost during the post-treatment follow up

*Abbreviations*: *LQ* Lower Quartile, *UQ* Upper Quartile

## Discussion

Concurrent infection with malaria and hookworm is common among people living in Kintampo North Municipality, and this may affect immune responses and clinical outcomes of these infections. The immune responses to hookworm infection may have a “bystander” effect on antimalarial immunity. In this study IgG1, IgG3 and IgM responses to the GMZ2 malaria vaccine candidate and constituent antigens were studied before and after anthelmintic treatment with albendazole.

Our result showed that the co-infected individuals had higher levels of IgG3 antibodies against GMZ2 and IgG1 against GLURP R0. These antibodies are known to be associated with protection against malaria [1, 23–25]. *Ascaris lumbricoides* co-infection with malaria were also found to show an increased anti-gametocyte immune response compared to the uninfected participants [4]. These findings suggest that the presence of the *N. americanus* worm may boost the antimalarial specific IgG1 and IgG3 antibody responses against malaria. This is supported by our previous findings that *P. falciparum* parasitaemia was lower in co-infected individuals compared to those with mono-infections [16]. However, our findings conflict with other helminth-malaria parasite co-infection study by Courtin et al. [26], who found *Schistosoma haematobium* infection led to significant reduction in *Plasmodium falciparum*-specific IgG responses levels directed to MSP-1 and to GLURP antigens. Another study in Zimbabwe, rather reported no association between Schistosoma infection and humoral response to malaria parasites [27]. Furthermore, Esen et al. [28], found significantly lower antibody response against GMZ2 and GLURP antigens after vaccination with the malaria vaccine candidate GMZ2 in individual who had *T. trichiura* present during vaccination. The observation from these studies indicate that helminths co-infection with *P. falciparum* may have varying antibody production against malaria antigens. These variations in the findings could be due to the differences in the biology and the anatomical position of the adult worms in the host, difference in the age of study participants and study design.

The study found no significant association with antibody response against MSP3 antigen in the co-infected individual, this corroborate with studies by M Esen, B Mordmuller, PM de Salazar, AA Adegnika, ST Agnandji, F Schaumburg, AB Hounkpatin, S Bruckner, M Theisen, S Belard, et al. [3], who reported no significant effect of *T. trichiura* on anti-MSP3 antibody concentration. Presumably, MSP3 may be the less immunogenic part of the vaccine [29, 30].

The study found a significantly positive association between the antibody levels (IgG3, and IgM) and age against GMZ2, and constituent antigens. This finding is consistent with studies by K Marsh, RH Hayes, DC Carson, L Otoo, F Shenton, P Byass, F Zavala and BM Greenwood [31] who reported a positive correlation between antibody titers and age, D Dodoo, A Aikins, KA Kusi, H Lamptey, E Remarque, P Milligan, S Bosomprah, R Chilengi, YD Osei and BD Akanmori [25] and B Adu, MK Cherif, S Bosomprah, A Diarra, FK Arthur, EK Dickson, G Corradin, DR Cavanagh, M Theisen, SB Sirima, et al. [18], also reported high levels of IgM against MSP3 and GLURP with age among Ghanaian children. These results are consistent with the hypothesis that immunity to malaria is largely effected through antibody-mediated mechanisms and that protective antibody levels to relevant antigens increase with age of an individual co-infected with other parasites [32]. The weak association observed between antibody levels and age in our study population may be due to the fact that most of the study participants were older and may have greater cumulative exposure to the malaria parasite with already developed acquired immunity compared to children [33].

The IgG1 and IgG3 antibody levels measured against GMZ2, were significantly decreased after albendazole treatment. Also, IgG3 response to GLURP R0 and IgG1 and IgM responses to MSP3 were significantly decreased after treatment. Helminth infections are usually associated with a predominantly Th2-type of immune response [34]. In our previous study, we observed an increased level of IL10 in *P. falciparum* and *N. americanus* co-infection, which significantly decreased after successful albendazole, treatment [16]. Since IL10 is known to provide help for B cells to produce antibodies [26], this may explain both the increased malarial antibodies in co-infected individuals and the decrease in antibody levels observed after hookworm treatment.

## Conclusion

This study revealed stronger antibody response against malaria vaccine candidate antigens (GMZ2 and GLURP R0) in the presence of *N. americanus* infection. However, treatment of hookworm/malaria co-infection resulted in a reduction in antibody responses against GMZ2 and constituent antigens. These findings require further assessment of hookworm/malaria co-infection on efficient treatment of malaria using malaria antigens as vaccine candidate.

## Data Availability

All data generated or analyzed during this study are included in this published article and can be requested from corresponding author.
